# Polarization-controlled chiral transport

**DOI:** 10.1038/s41377-025-01762-9

**Published:** 2025-02-10

**Authors:** Hang Zhu, Jian Wang, Andrea Alù, Lin Chen

**Affiliations:** 1 Wuhan National Laboratory for Optoelectronics and School of Optical and Electronic Information, Huazhong University of Science and Technology, Wuhan, 430074 China; 2 Photonics Initiative, Advanced Science Research Center, City University of New York, New York, NY 10031 USA; 3 Physics Program, Graduate Center, City University of New York, New York, NY 10016 USA; 4 Shenzhen Huazhong University of Science and Technology Research Institute, Shenzhen, 518063 China; 5 Key Laboratory of High Temperature Electromagnetic Materials and Structure of MOE, Wuhan University of Science and Technology, Wuhan, 430081 China

**Keywords:** Silicon photonics, Nanophotonics and plasmonics

## Abstract

Handedness-selective chiral transport is an intriguing phenomenon that not only holds significant importance for fundamental research but also carries application prospects in fields such as optical communications and sensing. Currently, on-chip chiral transport devices are static, unable to modulate the output modes based on the input modes. This limits both device functionality reconfiguration and information transmission capacity. Here, we propose to use the incident polarization diversity to control the Hamiltonian evolution path, achieving polarization-dependent chiral transport. By mapping the evolution path of TE and TM polarizations onto elaborately engineered double-coupled waveguides, we experimentally demonstrate that different polarizations yield controllable modal outputs. This work combines Multiple-Input, Multiple-Output, and polarization diversity concepts with chiral transport and challenges the prevailing notion that the modal outputs are fixed to specific modes in chiral transport, thereby opening pathways for the development of on-chip reconfigurable and high-capacity handedness-selective devices.

## Introduction

The wavelength, mode, and polarization multiplexing are recognized as the most effective solutions to address the need for multi-channel parallel communications in optical interconnects, optical computing, and optical sensing. Polarization, as an intrinsic degree of freedom of light, is playing an increasingly significant role in multitasking information transmission. Developing polarization channel devices is beneficial for enhancing the control of the optical degrees of freedom^1,2^, expanding information transmission capacity^3,4^, improving transmission efficiency^5^, and reducing channel crosstalk^6^. Successful implementations include polarization-controlled directional coupling^6^, valley-locked beam splitters^7^, circular polarization-dependent chiral ring resonators^2^, and multifunctional spin lasers^8^. These polarization channel devices provide valuable methods for designing reconfigurable optical routers and optical computing processors, thereby contributing to the development of programmable optical communication and optical computing network technologies.

In a different context, exceptional points (EPs), consisting of singularities that arise in non-Hermitian systems where eigenvalues and eigenvectors coalesce, have been raising significant interest in various physical disciplines, including electronics and photonics^9–14^. The introduction and control of gain and loss distributions in photonic systems have enabled researchers to study the fundamentals of EPs and the associated non-Hermitian phenomena in a wide range of optical systems, including microcavities^15–18^, waveguides^19–32^, gratings^33,34^, and photonic crystals^35,36^. Many intriguing phenomena, such as enhanced sensing^37–40^, unidirectional invisibility^41,42^, single-mode lasers^16,43^, and chiral dynamics^21–34^, have emerged due to the unique topological features around EPs, which not only are of importance from the fundamental standpoint but also have been leading to various cutting-edge photonic technologies. Out of the many associated phenomena, dynamically encircling an EP in non-Hermitian systems, has been under intense spotlight recently due to its chiral response, where the final system state depends on the handedness of EP encircling^21–34^. Chiral transport achieved in these optical systems shows significant potential applications, such as quantum computing, asymmetric optical switches^23^, polarization controllers^22,30^, optical isolators^44^, and more. To overcome the challenges of low chiral conversion efficiency and large device size, which limit the practical on-chip application of chiral transport devices, researchers have proposed to use Hamiltonian hopping to enhance chiral conversion efficiency^28^, and have explored methods involving moving EPs and fast encircling of EPs to reduce device size^31,33^. Moreover, previously reported chiral transport devices are static, with each output port locked to a specific mode regardless of the input, which limits functional reconfiguration and transmission capacity improvement.

In this article, we overcome the challenge that the output modes are fixed to specific modes independent of input modes in chiral transport by introducing polarization diversity in the EP-encircling dynamics. We show that polarization diversity can be used to control the evolution direction in the Hamiltonian parameter space, thereby achieving polarization-dependent state outputs. We present experimental results by mapping the evolution trajectories of TE and TM polarizations onto elaborately engineered double-coupled waveguides, showing different output modes corresponding to different polarizations at telecommunication wavelengths. Compared to previous research, our study challenges the inherent understanding that the output mode cannot be controlled by the input but is uniquely locked to a single mode in EP-encircling chiral transport, providing opportunities for the development of on-chip reconfigurable and high-capacity chiral transmission devices.

## Results

**Fig. 1:**
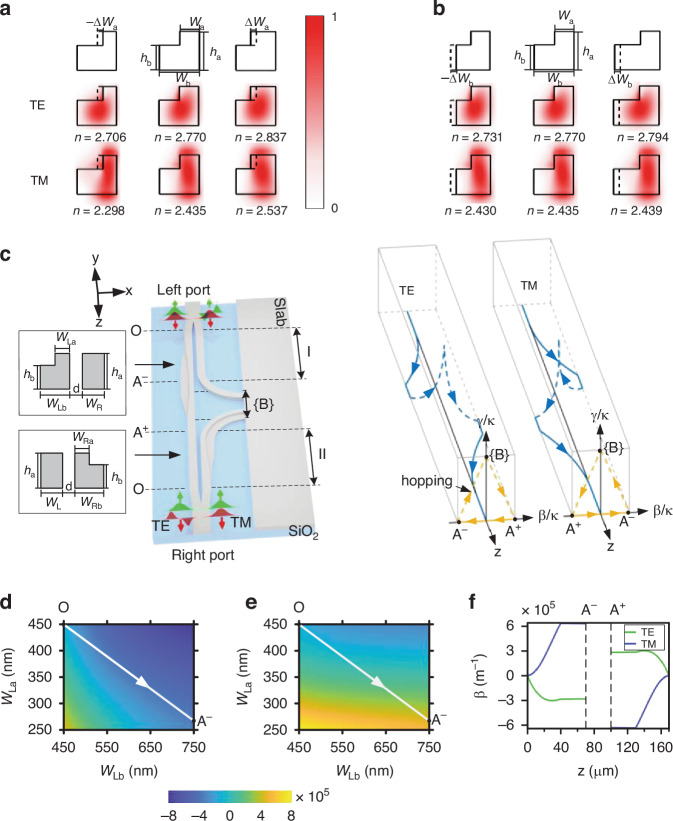
Double-coupled waveguides for polarization-controlled chiral transport. **a**, **b** Modal field and index variations for TE and TM polarizations by changing the upper (**a**) and lower (**b**) widths of the single L-shaped waveguide. Below the modal field distributions, *n* denotes the modal index. The lower and upper widths of the baseline waveguide are *W*_b_ = 700 nm and *W*_a_ = 350 nm, respectively, and their width variations are Δ*W*_a_ = Δ*W*_b_ = 100 nm. **c** Dual-coupled waveguides for demonstrating polarization-controlled chiral transport. The cross-sectional parameters are marked in the left panel. The blue line with arrows in the right panel shows the evolution trajectory, where its projection onto (*β*/*κ*, *γ*/*κ*) plane is marked by the brown line. **d**, **e** *β* versus *W*_La_ and *W*_Lb_ at 1550 nm for TE (**d**) and TM (**e**) polarizations, with *W*_R_ being fixed at 450 nm. The white straight lines represent the structural parameter variations from O to A^−^. **f** *β* as a function of the propagation distance, *z*. The entire silicon waveguides are covered by a 1-μm-thick SiO_2_ layer, with the refractive indices of Si and SiO_2_ being 3.478 and 1.444 at 1550 nm, respectively. The full height of the L-shaped waveguide is *h*_a_ = 340 nm, and the height of the lower waveguide is *h*_b_ = 220 nm. The modal field distributions in **a**, **b** are obtained by FDTD simulations

The evolution path around EPs has been conventionally realized using coupled dielectric waveguides of rectangular cross-section. In such systems, the output port of the co-designed chiral transport devices is locked to a specific mode regardless of the input incidence^21,23,28,31,33^. Changing the width of the rectangular waveguide influences the effective refractive indices of both TE and TM polarizations at the same time. Therefore, the associated Hamiltonian parameters undergo a co-directional evolution path for both polarizations. Here, instead we implement anti-directional evolution paths for TE and TM polarizations, achieved by introducing L-shaped waveguide cross-sections in double-coupled waveguides. We start by analyzing TE and TM modes supported by L-shaped silicon waveguides, as shown in Fig. 1a, b. It can be seen that TE and TM polarizations are distributed differently, where TE and TM polarizations are mainly distributed in the central and right regions of the waveguide, respectively. Although their effective refractive indices increase when either the top or bottom width increases, their sensitivity to the top and bottom widths differs. The TE polarization is insensitive to changes in the top width but sensitive to changes in the bottom width. Conversely, the TM polarization is sensitive to changes in the top width, but insensitive to changes in the bottom width. We are thus able to alter the top and bottom widths so that the effective refractive indices of TE and TM polarizations change in opposite directions. If an L-shaped waveguide and a rectangular waveguide are combined to form double-coupled waveguides, as shown in Fig. 1c, we can optimize the geometry such that the detuning of TE and TM polarizations change in opposite directions, resulting in polarization-controlled chiral transport. It should be noted that the detuning of TE and TM polarizations is proportional to the difference in their respective effective refractive indices. The evolution direction for the Hamiltonian parameters is determined by the detuning of double-coupled waveguides, rather than by the effective refractive index of a single waveguide. If only the width of either the top or bottom layer of the L-shaped waveguide is varied, the detuning for both TE and TM polarizations will change in the same direction, which cannot achieve the goal of polarization-controlled chiral transport. In this work, the device length for the double-coupled waveguides is chosen as 170 µm to strike a balance between satisfying the adiabatic condition and minimizing device length. More detailed optimization of the geometrical parameters of the double-coupled waveguides can be found in Supplementary Note 1.

The double-coupled waveguides can be rigorously described with the evolution equation as

1 $$i\partial /\partial z|\psi \rangle =H|\psi \rangle$$

where the eigenfunction is expressed as $|\psi (z)\rangle ={[{a}_{1}(z),{a}_{2}(z)]}^{T}$, *a*_1_(z) and *a*_2_(z) are the amplitudes of modes in each waveguide, and the eigenstates [1,1]^T^ and [1,−1]^T^ correspond to the symmetric and anti-symmetric modes, respectively. The Hamiltonian can be written as

2 $$H(z)=\left[\begin{array}{cc}\beta (z)+i\gamma (z) & \kappa (z)\\ \kappa (z) & -\beta (z)\end{array}\right]$$

Here, *β*(z), *γ*(z), and *κ*(z) represent the degree of detuning, loss rate, and coupling strength of the system, respectively. The Hamiltonian parameters (*β*, *γ*, and *κ*), associated with the waveguide geometry, can be theoretically retrieved based on coupled mode theory^45^ and the Beer–Lambert–Bouguer law^46^. The two eigenvalues are ${E}_{1,2}={i}{\gamma} /{2} {\pm} \sqrt{{\kappa}^{2}+{({\beta} +{i}{\gamma} /{2})}^{2}}$ and the associated eigenvectors $|{X}_{1,2}\rangle ={[\sqrt{1{\pm}M},{\pm} \sqrt{1-({\pm} M)}]}^{T}/\sqrt{2}$, where $M=(\beta +i\gamma /2)/\sqrt{{\kappa }^{2}+{(\beta +i\gamma /2)}^{2}}$, indicating that the system has an EP at (*β*/*κ, γ*/*κ*) = (0,2). Previous studies have shown that when the dynamic Hamiltonian trajectories surround the EP, the different losses experienced by different eigenstates lead to the generation of nonadiabatic transitions (NAT), which makes the output modes depend on the handedness of EP encircling^10,21,23,28,33^. In Section I, the width of the right rectangular waveguide is kept constant, and the top and bottom widths of the left L-shaped waveguide reduces and increases along the *z* direction. In this situation, the detuning of TE and TM polarizations between the two waveguides is negative and positive, respectively, as shown in Fig. 1d–f. In Section II, the positions of the rectangular and L-shaped waveguides are swapped compared to Section I. Therefore, the detuning of TE and TM polarizations are opposite to those in Section I, respectively. To avoid path-dependent loss in encircling EPs, we have employed the strategy of “Hamiltonian hopping” between convergent eigenstates at Hamiltonian boundaries^28^, i.e., points A^−^, {B}, and A^+^, as indicated in Fig. 1c. A^−^ (A^+^) can be reached by increasing the gap distance, *d*, which effectively makes *κ*(z) → 0. It should be noted that TE and TM polarizations take opposite values at A^−^ (A^+^) points, due to their opposite detuning. A large loss rate *γ* at {B} can be implemented using a semi-infinite slab waveguide to replace the first waveguide. Bend waveguides are used to connect the right waveguide with the slab waveguide. Once the guided waves reach the slab waveguide through the bend, light is not reflected back, i.e., *γ*/*κ* → ∞ as required at {B}. It should be emphasized that in previous studies using the *Hamiltonian hopping* strategy with coupled dielectric waveguides of rectangular cross-section, chiral mode switching was achieved by Hamiltonian transitions between convergent eigenstates at Hamiltonian boundaries during EP encircling^28^. However, in these studies, the evolution directions for TE and TM polarizations were the same, preventing the realization of polarization-dependent chiral switching. In contrast, the present work employs an intricately designed L-shaped waveguide structure. This design leverages the convergent eigenstates at Hamiltonian boundaries, enabling Hamiltonian transitions between these states during EP encircling, thereby achieving chiral mode switching. Additionally, it facilitates polarization-dependent chiral switching, as TE and TM polarizations evolve in opposite directions (Fig. 1c). In contrast, previous studies on chiral mode switching using enclosed trajectories encircling EPs were unable to implement reverse evolution directions for TE and TM polarizations, which prevented polarization-dependent chiral switching^23,26,31,33^. More details regarding the dependence of *γ* and *κ* on the geometry of the double-coupled waveguides can be found in Supplementary Note 1.

**Fig. 2:**
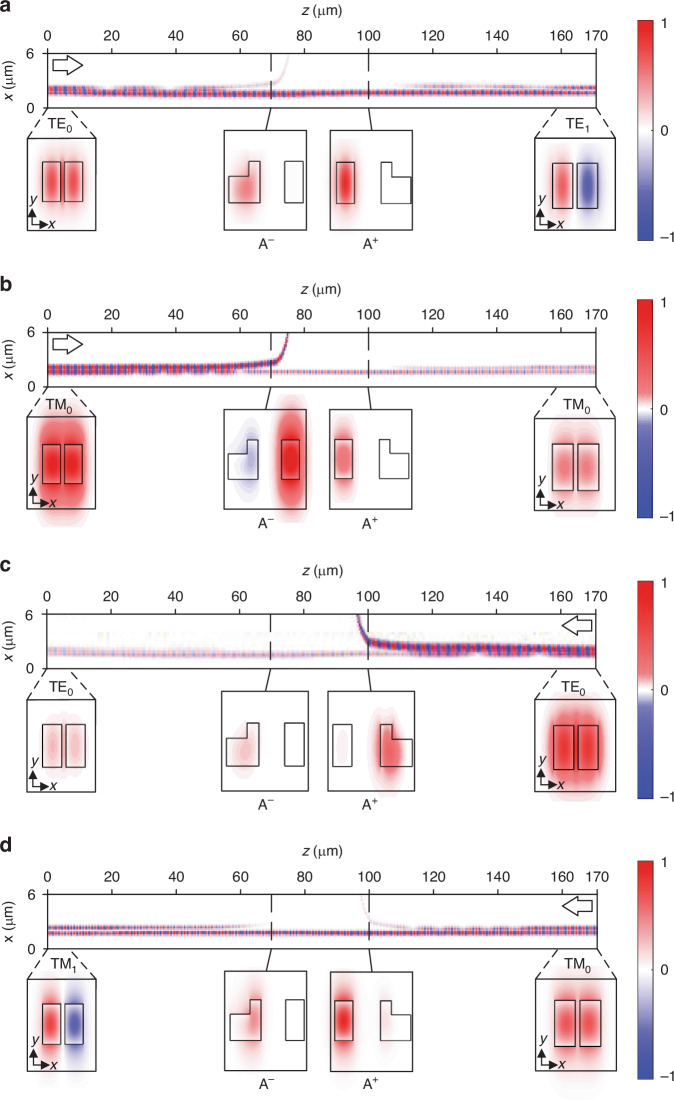
Simulated field distributions. **a**, **c** Field distributions of *E*_x_, when TE_0_ mode inputs from the left (**a**) and right (**c**) ports, respectively. **b**, **d** Field distributions of *E*_y_, when TM_0_ mode inputs from the left (**b**) and right (**d**) ports, respectively. The simulated field distributions in the double-coupled waveguides at *z* = 0, 70, 100, and 170 µm are obtained by FDTD simulations

To validate the functionality of polarization-controlled chiral transport, we conducted full-wave simulations using the finite-difference time-domain (FDTD) method to simulate the field intensity distributions of the double-coupled waveguides (Fig. 2). The output mode is not locked to a specific mode, but it is dependent on the incident polarization. For the left (right) port input with TE_0_ and TM_0_ modes, the output mode is TE_1_ (TE_0_) and TM_0_ (TM_1_) modes, respectively. When the TE_0_ mode is input from the left port, most of the light energy resides in the left waveguide between A^−^ and A^+^, where the dominant eigenstate is [0,1]^T^ (Fig. 2a). However, when TM_0_ mode is input, most of the light energy is concentrated in the right waveguide at A^−^ and eventually dissipates as it travels through the bent waveguide to the slab waveguide, as shown in Fig. 2b. When the TE_0_ (TM_0_) mode is input from the right port, the evolution process is similar to the one when TM_0_ (TE_0_) mode is input from the left port, and the output mode will be TE_0_ (TM_1_) mode (Fig. 2c, d). It should be noted that the output mode is locked to TE_1_ (TM_0_) for the left port input and to TE_0_ (TM_1_) for the right port input, regardless of whether the input is a symmetric or antisymmetric mode. Simulation results with anti-symmetric mode incidence can be found in Supplementary Note 2. Overall, the simulation results demonstrate polarization-controlled chiral transmission for the double-coupled waveguides.

To understand the physics behind the intriguing polarization-controlled chiral response, the light transmission in the double-coupled waveguides can be rigorously described by Eq. (1). Assuming that *H*(z) remains constant over the distance interval [z_0_,z], the final state can be written as

3 $$|\psi (z)\rangle ={c}_{1}({z}_{0}){e}^{i{E}_{1}(z-{z}_{0})}{X}_{1}+{c}_{2}({z}_{0}){e}^{i{E}_{2}(z-{z}_{0})}{X}_{2}$$

**Fig. 3:**
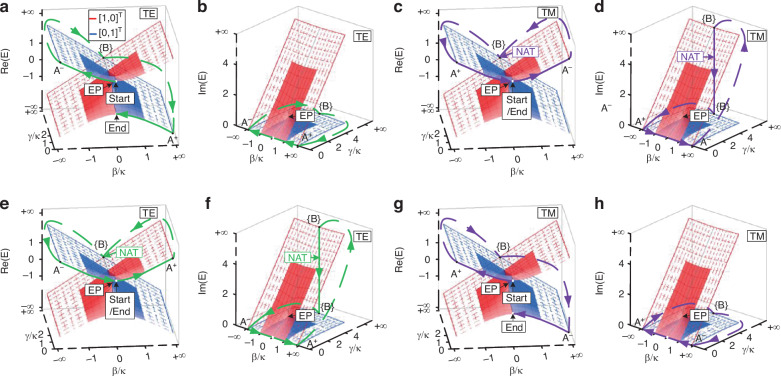
System states evolving on the Riemann surfaces. The evolution trajectories in the Riemann surfaces are formed by the real part Re(*E*) and imaginary part Im(*E*) of the energy spectra of *H*, when the symmetrical mode is input from the left (**a**–**d**) and right (**e**–**h**) ports, respectively. The dashed meshes represent the Hamiltonian parameters extended to infinity. The blue and red solid lines represent the parameter space boundary, associated with the eigenstates as [0,1]^T^ and [1,0]^T^, respectively. The dashed lines refer to Hamiltonian hopping among A^−^, {B} and A^+^

The initial state is $|\psi ({z}_{0})\rangle ={c}_{1}({z}_{0}){X}_{1}+{c}_{2}({z}_{0}){X}_{2}$ at z_0_. Equation (3) indicates that the real and imaginary parts of the eigenvalues affect the phase and magnitude, respectively. We can thus present the dynamic Hamiltonian trajectories in the Riemann surfaces formed by the energy spectra of Hamiltonian for TE and TM polarizations, as schematically shown in Fig. 3. The dynamic trajectories are opposite for TE and TM polarizations when they are injected into the same port, due to the opposite sign of the detuning *β* of TE and TM polarizations.

For symmetrical modes injected from the left port, the associated state [1,1]^T^ at the starting point (*β*/*κ, γ*/*κ*) = (0, 0) is situated in the upper half of the Riemann surface (Fig. 3a–d). When a TE_0_ mode is injected and evolves clockwise from (0, 0) to A^−^, *X*_1_ is dominant, and it suffers from low loss as the imaginary part of *E*_1_ is zero. The Hamiltonian ultimately returns to (0, 0) after it undergoes successive hopping between A^−^, {B} and A^+^. Throughout the entire process, *X*_1_ is always dominant, resulting in the output state *X*_1_ = [1,−1]^T^ on the lower half of the Riemann surface, corresponding to the TE_1_ mode (Fig. 3a, b). In contrast to the TE_0_ mode, the Hamiltonian evolves oppositely when the TM_0_ mode is injected. During the evolution process from (0, 0) to A^−^, the Hamiltonian evolves anticlockwise, in which *X*_2_ is dominant, and *X*_1_ is slightly excited since the adiabatic condition cannot be strictly satisfied. During the hopping between A^−^, {B}, and A^+^, the dominant state *X*_2_ incurs significant loss and eventually is dissipated, while *X*_1_ remains lossless and becomes the dominant state, i.e., a NAT occurs. The output state returns to *X*_1_ = [1,1]^T^ at (0, 0) on the upper half of the Riemann surface, corresponding to the TM_0_ mode (Fig. 3c, d).

For symmetrical modes injected from the right port, the initial state [1,1]^T^ is also situated in the upper half of the Riemann surface (Fig. 3e–h). For TE_0_ injection, the dominant eigenstate transitions from *X*_2_ to *X*_1_, when the system experiences NAT. The system state becomes [1,1]^T^ at the terminal point (0, 0), corresponding to TE_0_ mode, as it evolves along the upper surface of the Riemann surface (Fig. 3e, f). For TM_0_ injection, *X*_1_ is consistently dominant, and the system state finally outputs as [1,−1]^T^ at (0, 0), corresponding to TM_1_ mode (Fig. 3g, h). The dynamics process for the injection of symmetrical modes of different polarizations further validates the polarization-controlled chiral transport of the double-coupled waveguides. It should be noted that the output modes are locked with the same polarization incidence, regardless of the mode order. More details regarding the dynamics process can be found in Supplementary Note 3.

**Fig. 4:**
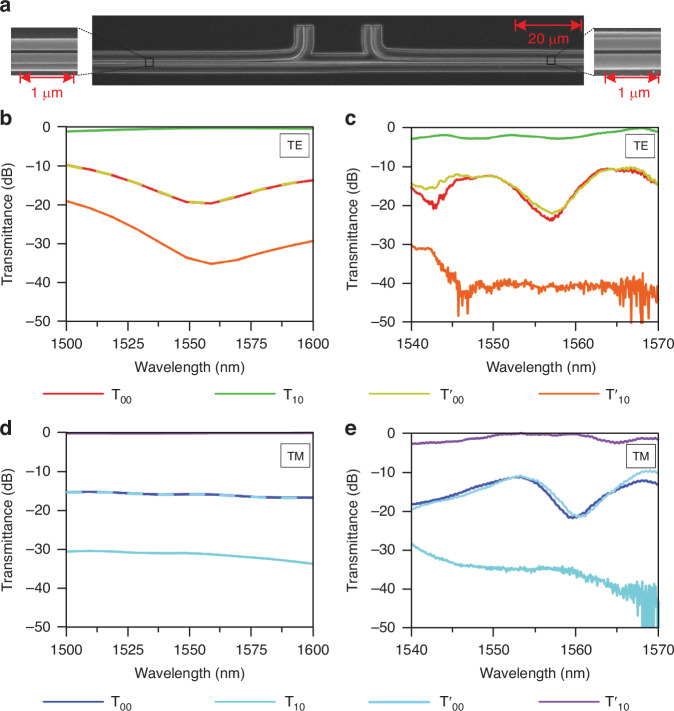
Experimental demonstration. **a** SEM image of the device. **b**, **d** Simulated transmittance spectra at the output port over the wavelength range of 1500–1600 nm with TE_0_ (**b**) and TM_0_ (**d**) injection. **c**, **e** Experimental transmittance spectra at the output port over the wavelength range of 1540–1570 nm with TE_0_ (**c**) and TM_0_ (**e**) injection

Scanning electron microscope (SEM) images for the double-coupled silicon waveguides in one of the fabricated samples are shown in Fig. 4a. The zoomed-in images on the left and right planes represent the regions bounded by the black rectangles (see Supplementary Note 4 for the fabrication details and transmission measurement scheme). These images clearly indicate that the L-shaped and rectangular waveguides are reversed in the regions near the left and right ports. The simulated and measured transmission efficiencies for TE_0_ and TM_0_ input around 1550 nm are shown in Fig. 4b–e. *T*_mn_ (*T*'_mn_) represents the transmission efficiency of TE_m_ or TM_m_ mode that outputs from the right (left) port when TE_n_ or TM_n_ mode inputs from the left (right) port. When TE_0_ (TM_0_) mode is injected from the left port, the extracted experimental efficiency deviation at 1550 nm is *T*_10_–*T*_00_ ≈ 12 dB (*T*_00_–*T*_10_ ≈ 13 dB), i.e., *T*_10_ ≫ *T*_00_ (*T*_00_ ≫ *T*_10_), indicating that TE_1_ (TM_0_) mode dominates in the output. In contrast, when TE_0_ (TM_0_) mode is injected from the right port, we have *T*'_00_–*T*'_10_ ≈ 28 dB (*T*'_10_–*T*'_00_ ≈ 22 dB), i.e., *T*'_00_ ≫ *T*'_10_ (*T*'_10_ ≫ *T*'_00_), indicating that the output mode is dominated by TE_0_ (TM_1_) mode. It should be emphasized that mode crosstalk in coupled waveguides cannot be completely avoided, as the adiabaticity condition cannot be strictly fulfilled^23^. From a device performance perspective, increasing the device length can help reduce crosstalk, but this also increases fabrication complexity and device size, which may hinder high-density photonic integration. We have demonstrated that mode crosstalk can be further minimized by doubling the device length (see Fig. S6 in Supplementary Note 3). It is important to note that the use of Hamiltonian hopping does not offer a significant advantage over previous strategies in terms of reducing mode crosstalk. In fact, the crosstalk observed in our device is comparable to that in devices that do not use Hamiltonian hopping^21,23,24,28,31^. These measurement results are consistent with the polarization-controlled chiral transport as has been predicted by the aforementioned theory and simulation. The experimental results generally match the simulation results, with some deviations mainly attributed to fabrication errors in the sample and testing inaccuracies due to noise, especially when the transmission efficiency is very low. Specifically, the testing errors primarily arise from alignment errors and the limitation of the single writing field area during the EBL fabrication process of the L-shaped waveguides, as well as the impact of dark current noise in the spectrometer on low transmission efficiency.

If further developed to guide different output polarizations along different waveguides, the polarization-controlled chiral converter can be extended to construct a chiral polarization router. This holds promise for applications in quantum walk systems to generate spin-correlated quantum states that are insensitive to input modes, indicating significant potential in quantum information processing^47^. For example, polarization-switchable logic gates can be realized by using polarization-controlled chiral converters integrated with Y-branch waveguides. More sophisticated polarization-switchable logic gate functions are expected to emerge by combining the polarization-controlled chiral converters with other complex logic gates^48^. However, some logic gate designs are limited by strong polarization dependence. Therefore, to advance the development of sophisticated polarization-switchable logic gates using polarization-controlled chiral converters, it is crucial to address the polarization-dependence issue present in existing optical logic gates. Moreover, selectively introducing gain or loss in polarizations might be beneficial to achieve on-chip polarization-controlled output from lasers^49^. Compared to previous studies on chiral transport based on EP encircling^23–29^, we have also introduced the degree of polarization freedom to increase the number of photonic transmission channels, which helps enhance multiplexing dimensions and expand communication capacity. Simulation results indicate that even with a ±50 nm alignment error, our device can maintain crosstalk below −10 dB, exhibiting favorable polarization-dependent asymmetric transmission effects. The device exhibits high fabrication robustness, facilitating its future practical fabrication and applications. The detailed simulation results are provided in Supplementary Note 5. In previous studies on chiral transmission, the chiral transmission efficiency is defined as the maximum transmittance across all input states and encircling directions^31^. The transmission efficiency of the proposed polarization-controlled chiral devices approaches unity, owing to the use of Hamiltonian hopping, which avoids path-dependent losses. Most studies rely on the strategy of encircling EPs to achieve chiral transmission, but this approach suffers from path-dependent losses along the entire evolution path, leading to a low chiral transmission efficiency. The maximum reported chiral transmission efficiency is below 0.46^21,23,24,33^. Other strategies involve slow evolution without encircling near EPs, which not only suffer from path-dependent losses along the entire evolution path but also induce additional NAT for both evolution directions^50,51^. As a result, the chiral transmission efficiencies in these cases are even lower than those achieved using the strategy of encircling near EPs.

It should be emphasized that, low transmission efficiency in the NAT-associated directions can limit the degree of freedom in multiplexing. Although NAT can, in principle, be achieved by applying either loss or gain, existing experiments have predominantly relied solely on loss to implement NAT in passive photonic devices^21,23,24,26,28–34^. This preference stems from the fact that loss is easier to implement experimentally than gain. In previous studies on chiral transmission based on double-coupled waveguides without considering polarization multiplexing, only one branch of high transmission was achieved, corresponding to one single degree of freedom for multiplexing. In contrast, the proposed polarization-controlled chiral transport devices can utilize two branches of high transmission for polarization multiplexing, enabling polarization multiplexing and corresponding to two degrees of freedom. To further enhance the degrees of multiplexing, one potential approach is to implement multiple groups of chiral mode switching or to replace the current passive devices with active ones to improve transmission efficiency. While we have demonstrated the implementation of polarization diversity using a second-order system with chiral transport, polarization diversity in third-order or higher-order systems could be achieved by using three or more L-shaped coupled waveguides in principle. As an example, we have numerically demonstrated chiral transport for polarization diversity in a third-order Hamiltonian system with three-coupled L-shaped waveguides. Further details can be found in Supplementary Note 6.

## Discussion

In conclusion, we have reported an on-chip polarization-controlled reconfigurable chiral transport device. We have shown that, in a double-coupled waveguide system, the Hamiltonian can be controlled to evolve around EPs in opposite directions for TE and TM polarizations, achieving polarization-controllable chiral transmission. Unlike previous works where output modes were uniquely determined by the incident direction, our approach allows output modes to be controlled by the incident polarization states. The silicon photonic experiments have verified the polarization-controlled chiral transmission effect. Our approach is general and can be extended to chiral transport in other physical fields such as acoustics, electronics, and condensed matter physics. From an application perspective, this novel device can function as a routing unit for high-performance optical communication and optical computing networks, effectively increasing optical information transmission capacity and enhancing optical computing power.

## Materials and methods

### Fabrications

The fabrication of the devices is combined with three-step electron-beam lithography (EBL), inductively coupled plasma (ICP) etching, electron-beam evaporation (EBE), and plasma-enhanced chemical vapor deposition (PECVD). The first step, EBL and EBE, is aimed at forming the Au marks on an SOI wafer for alignment. The second step, EBL and ICP, is used to define the partially etched layer of the L-shaped silicon waveguides. The fully-etched layer of the L-shaped silicon waveguides is fabricated by the third-step EBL and ICP. Finally, the PECVD is applied to deposit a 1-μm-thick SiO_2_ cladding layer to cover the entire device.

### Measurements

An amplified spontaneous emission (ASE) source (OVLINK ASE-CL-20-B) provides the near-infrared light, and its polarization is adjusted by a polarizing beam splitter (PBS) and a polarization controller. The grating coupler is used to couple the light from the fiber into TE_0_ mode or decouple the TE_0_ mode out of the silicon waveguide back into the fiber. The decoupled light will be collected by the optical power meter (PMSII-A) and a spectrometer (YOKOGAWA AQ6370C). See more details on the measurements in Supplementary Note 4.

## Supplementary information

Supplementary Information for Polarization-Controlled Chiral Transport

## Data Availability

All data required to interpret the results in this paper are provided within the main text and supplementary material. Any additional data in this study are available from the corresponding authors upon request. The code that supports the plots within this paper is available from the corresponding authors upon request.
