# Supplementary material for: Polarization-controlled chiral transport
Source: Light Sci Appl. 2025 Feb 10;14:77. doi: 10.1038/s41377-025-01762-9 (PMC11808067; doi:10.1038/s41377-025-01762-9)
Supplement: Supplementary file 1 — Supplementary Information for Polarization-Controlled Chiral Transport [file 41377_2025_1762_MOESM1_ESM.docx]

**Supplementary Information for**

**Polarization-Controlled Chiral Transport**

Hang Zhu^1^, Jian Wang^1^, Andrea Alù^2,3^ and Lin Chen^1,4,5,*^

^1^ Wuhan National Laboratory for Optoelectronics and School of Optical and Electronic Information, Huazhong University of Science and Technology, Wuhan 430074, China

^2^ Photonics Initiative, Advanced Science Research Center, City University of New York, New York, NY 10031, USA

^3^ Physics Program, Graduate Center, City University of New York, New York, NY 10016, USA

^4^ Shenzhen Huazhong University of Science and Technology Research Institute, Shenzhen 518063, China

^5^ Key Laboratory of High Temperature Electromagnetic Materials and Structure of MOE, Wuhan University of Science and Technology, Wuhan 430081, China

* Corresponding author’s email: [chen.lin@mail.hust.edu.cn](mailto:chen.lin@mail.hust.edu.cn)

**Supplementary Note 1: Structural and Hamiltonian parameters**


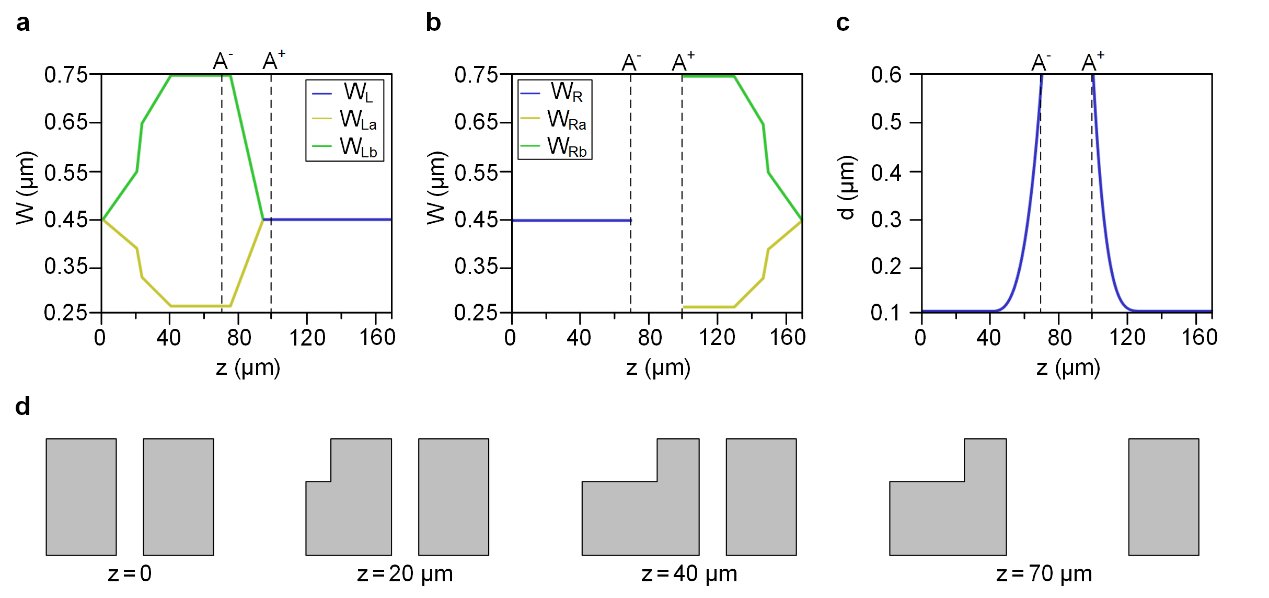


**Fig. S1 | Structural parameters of the double-coupled waveguides.** **a-c** The left (**a**) and right (**b**) waveguide widths, and gap distance (**c**) versus z. **d** Schematic illustration of the double--coupled waveguides at different positions.

The dependence of W_L_ (W_La_/W_Lb_), W_R_ (W_Ra_/W_Rb_) and d on z is depicted in Figs. S1a-c, respectively. Figure S1d shows the schematic of the waveguide profiles at different positions along the propagation direction in Section Ⅰ. In Section II, the positions of the rectangular and L-shaped waveguides are swapped compared to Section I.

On one hand, it is crucial to control the variation of the structural parameters to ensure that the entire evolution process satisfies the adiabatic condition, thereby allowing the desired output mode to dominate. On the other hand, if the structural parameters change too slowly, the adiabatic condition can be effectively maintained, but the device becomes excessively long, which is undesirable for high-density integration. As a result, it is necessary to strike a balance between satisfying the adiabatic condition and minimizing device length when designing the double-coupled waveguides.

For Section I (O → A^-^), the chosen lengths for the four segments within the 0–70 µm range are 20 µm, 5 µm, 15 µm, and 30 µm. The width of the first three segments changed linearly with propagation distance, as shown in Fig. S1a. It seems that, the width variation in the second segment shows a sudden jump, which results from a significantly higher growth rate of the width change compared to the first and third segments. In the fourth segment, the width is constant, but the gap distance is increased. It is well known that in coupled waveguides, mode coupling tends to occur when the detuning is small or the coupling strength is large. Therefore, the structural parameters were carefully designed, taking both detuning and coupling strength into account, as illustrated in Fig. 1f of the main text and Fig. S2a.

Let us first consider the TM polarization. In the first segment, the average detuning is smaller than that in the second and third segments. Therefore, we set the segment length to 20 µm, which is sufficiently long to prevent mode coupling. In the second segment, the detuning is already large, and the coupling strength is relatively small. As a result, a small segment length of 5 µm is adequate to avoid mode coupling without requiring slow parameter variation. In the third segment, the coupling strength increases compared to the second segment. To address this, we set a longer segment length of 15 µm to prevent mode coupling. The coupling strength at the beginning of the fourth section is already very large. To prevent mode coupling and minimize the device length, we increase the gap distance from 0.1 µm to 0.6 µm, which significantly lowers the coupling strength. As a result, the length for the fourth segment is shortened to 30 µm.

Next, we consider the TE polarization. Similar to the TM polarization, the first segment has a smaller average detuning compared to the second, third and fourth segments. A segment length of 20 µm is sufficient to avoid mode coupling. For the second, third, and fourth segments, the detuning is large, and the coupling strength remains relatively small. The chosen segment lengths of 5 µm, 15 µm, and 30 µm are sufficient to ensure adiabatic mode evolution.

For Section II (O → A^+^), we have employed the same design strategy as that in Section I to strike a balance between satisfying the adiabatic condition and minimizing device. The chosen lengths for the four segments within the 100–170 µm range are 30 µm, 15 µm, 5 µm, and 20 µm.

*κ*, *γ* and the effective refractive index of each waveguide versus z is shown in Fig. S2.


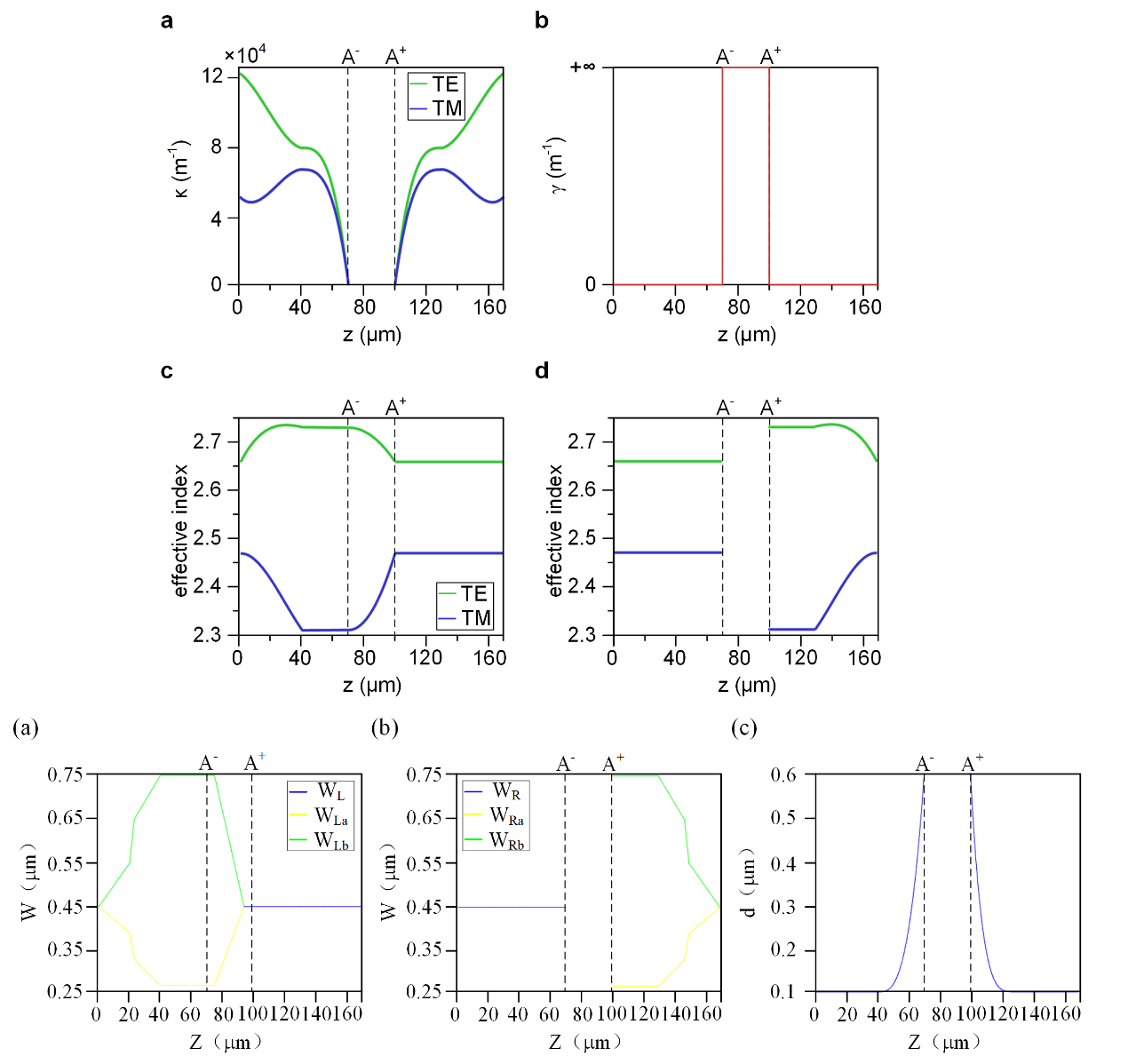


**Fig. S2 | The corresponding Hamiltonian parameters variation.** **a**, **b** κ (**a**) and γ (**b**) as a function of the propagation distance, z. **c**, **d** The effective refractive index of TE and TM polarizations as a function of the propagation distance, z, for the left (**c**) and right (**d**) waveguides.

**Supplementary Note 2: Simulated results with anti-symmetric mode incidence**


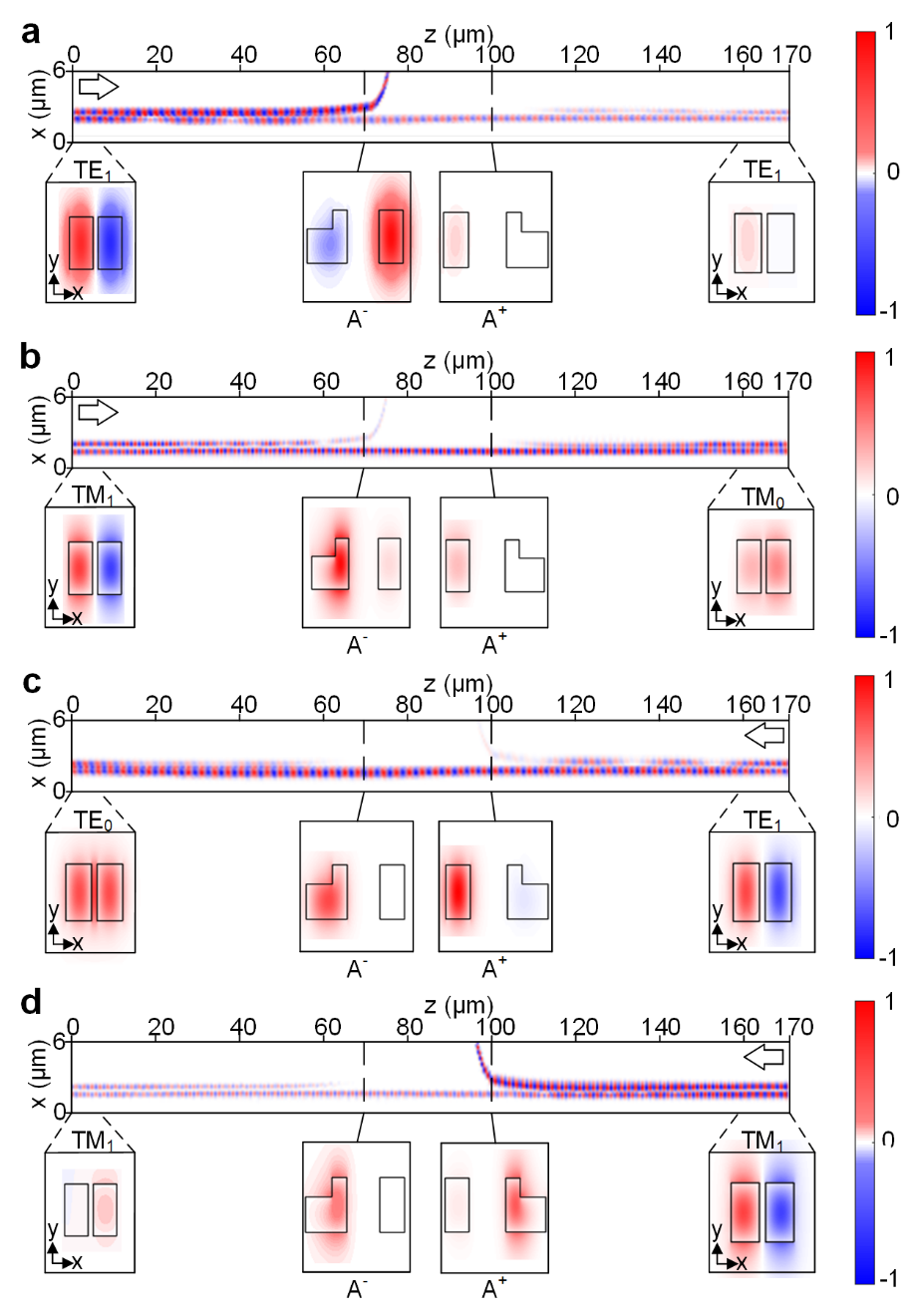


**Fig. S3 | Simulated field distributions.** **a**, **c** Field distributions of E_x_, when TE_1_ mode inputs from the left (**a**) and right (**c**) ports, respectively. **b**, **d** Field distributions of E_y_, when TM_1_ mode inputs from the left (**b**) and right (**d**) ports, respectively.

**Supplementary Note 3: Dynamic process**


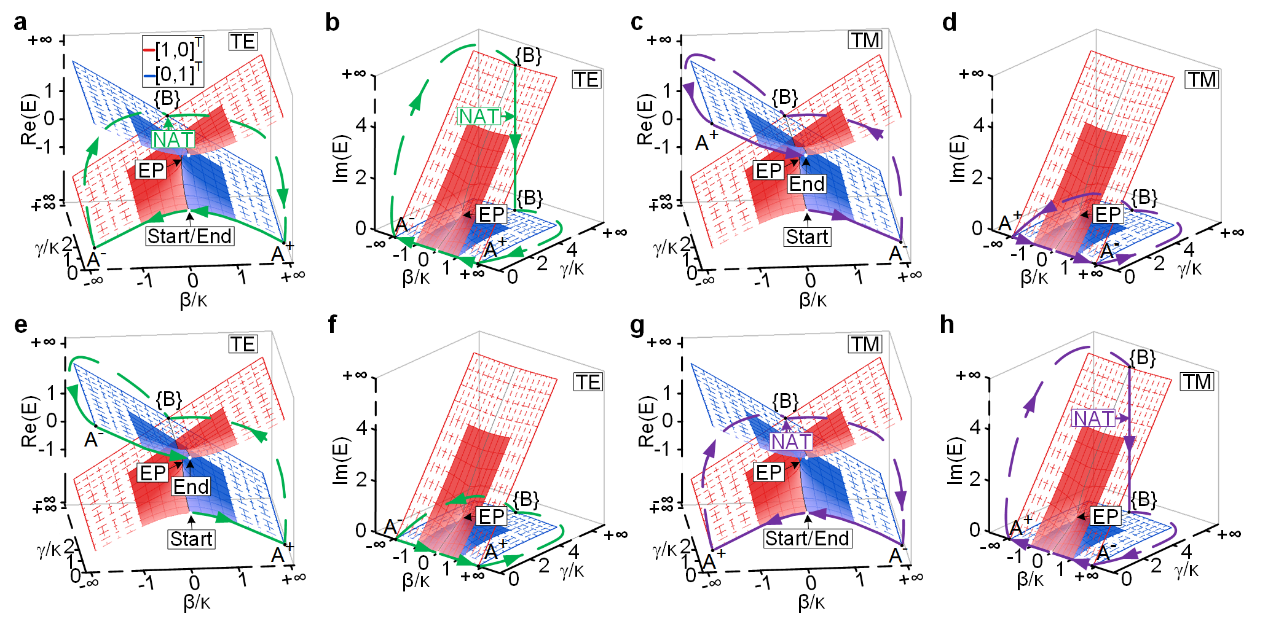


**Fig. S4 | System states evolving on the Riemann surfaces.** The evolution trajectories in the Riemann surfaces formed by the real part Re(*E*) and imaginary part Im(*E*) of the energy spectra of *H*, when the anti-symmetrical mode is input from the left (**a**-**d**) and right (**e**-**h**) ports, respectively.

For anti-symmetrical modes injected from the left port, the initial state $\left[1,-1\right]^{T}$ at the starting point $\left(\beta/\kappa,\gamma/\kappa\right)=\left(0,0\right)$ is situated in the lower half of the Riemann surface (Figs. S4a-d). When TE_1_ mode is injected and evolves clockwise from $\left(0,0\right)$ to A^-^, $X_{2}$ is dominant and the other state $X_{1}$ is slightly excited. Because the dominant state $X_{2}$ incurs significant loss during “Hamiltonian hopping” from A^-^ to {B}, it dissipates entirely. Meanwhile, $X_{1}$ remains lossless and becomes dominant from {B} to A^+^, i.e., NAT occurs. The output state returns to $X_{1}=\left[1,-1\right]^{T}$ at $\left(0,0\right)$ in the lower half of the Riemann surface, corresponding to TE_1_ mode (Figs. S4a, b). In contrast to TE_1_ mode, the Hamiltonian evolves oppositely when TM_1_ mode is injected. During the evolution process from $\left(0,0\right)$ to A^-^, $X_{1}$ is dominant since it suffers from low loss as the imaginary part of $E_{1}$ is zero. After the Hamiltonian successively experiences hopping between A^-^, {B}, and A^+^, it ultimately returns to $\left(0,0\right)$. Throughout the entire process, $X_{1}$ is consistently dominant, resulting in an output state of $X_{1}=\left[1,1\right]^{T}$ on the upper half of the Riemann surface, corresponding to TM_0_ mode (Figs. S4c, d).

For anti-symmetrical modes injected from the right port, the initial state $\left[1,-1\right]^{T}$ is also situated in the upper half of the Riemann surface (Figs. S4e-h). For TE_1_ injection, $X_{1}$ is consistently dominant, and eventually evolves along the upper surface of the Riemann surface to become $\left[1,1\right]^{T}$ at $\left(0,0\right)$, corresponding to TE_0_ mode (Figs. S4e, f). For TM_1_ injection, the dominant eigenstate transitions from $X_{2}$ to $X_{1}$, due to the occurrence of NAT, and eventually evolves along the lower surface of the Riemann surface to become $\left[1,-1\right]^{T}$ at $\left(0,0\right)$, corresponding to TM_1_ mode (Figs. S4g, h).


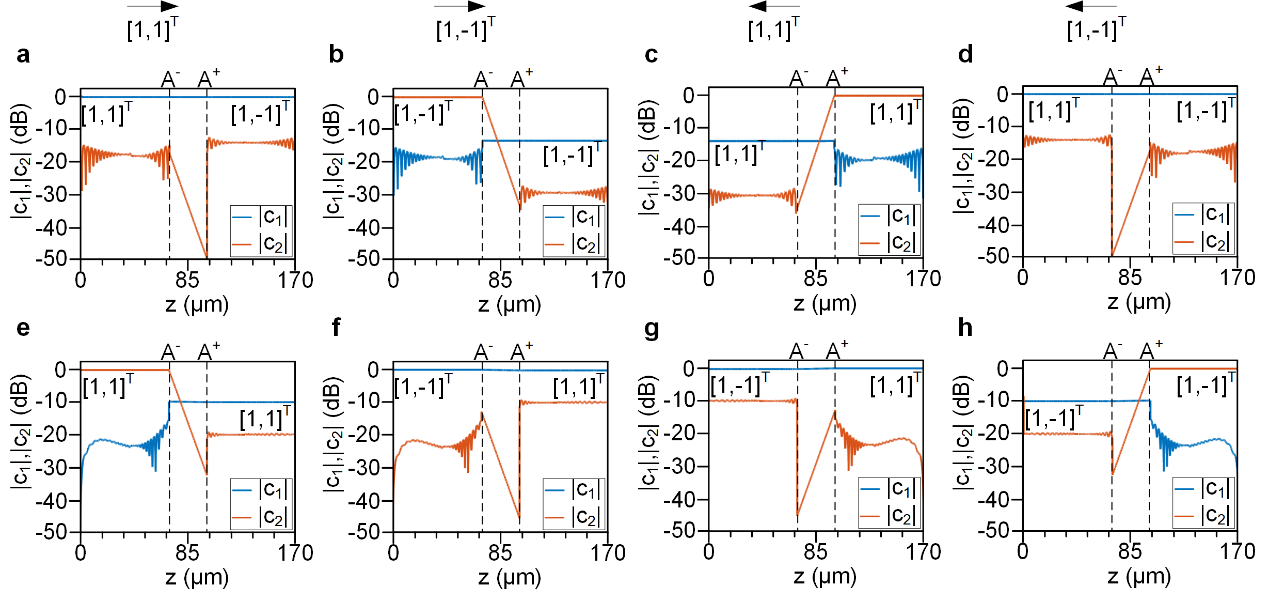


**Fig. S5 | Dynamics of the evolution trajectories for TE and TM polarizations.** |c_1_| and |c_2_| represent the amplitude of the two eigenstates, $|X_{1}\rangle$ and $|X_{2}\rangle$. Coefficients |c_1_| and |c_2_| versus the propagation distance, z, for (**a**-**d**) TE and (**e**-**h**) TM polarizations. The left-pointing and right-pointing arrows indicate the injection of left and right ports, respectively.

In order to validate the chiral dynamics described in Fig. 3 in the main text, the required Hamiltonian parameters (Fig. 1f and Fig. S2) are chosen to enable the evolution trajectories. Figure S5 illustrates the evolution of TE (a-d) and TM (e-h) modes, respectively. For TE polarization, the initial state is [1,1]^T^ when it inputs from the left port. The dominant eigenstate is always the eigenstate, $|X_{1}\rangle$, and the final state is [1,-1]^T^ (Fig. S5a). When [1,1]^T^ inputs from the right port, the dominant eigenstate is $|X_{2}\rangle$ for z > A^+^, and shifts to $|X_{1}\rangle$ in the distance interval between A^+^ and A^-^ due to the emergence of NAT, and the final state returns to [1,1]^T^ (Fig. S5c). When [1,-1]^T^ inputs from the left port, the finial state is [1,-1]^T^ due to the emergence of NAT (Fig. S5b). When [1,-1]^T^ inputs from the right port, the finial stat is [1,1]^T^ (Fig. S5d).

For TM polarization, the initial state is [1,1]^T^ when it inputs from the left port. The dominant eigenstate is $|X_{1}\rangle$ for z > A^-^, and shifts to $|X_{2}\rangle$ in the distance interval between A^-^ and A^+^ due to the emergence of NAT, and the final state returns to [1,1]^T^ (Fig. S5e). When [1,1]^T^ inputs from the right port, the dominant eigenstate is always the eigenstate, $|X_{2}\rangle$, and the final state is [1,-1]^T^ (Fig. S5g). When the initial state [1,-1]^T^ inputs from the left port, the finial state is [1,1]^T^ (Fig. S5f). When it inputs from the right port, the finial stat is [1,-1]^T^ due to the emergence of NAT (Fig. S5h).


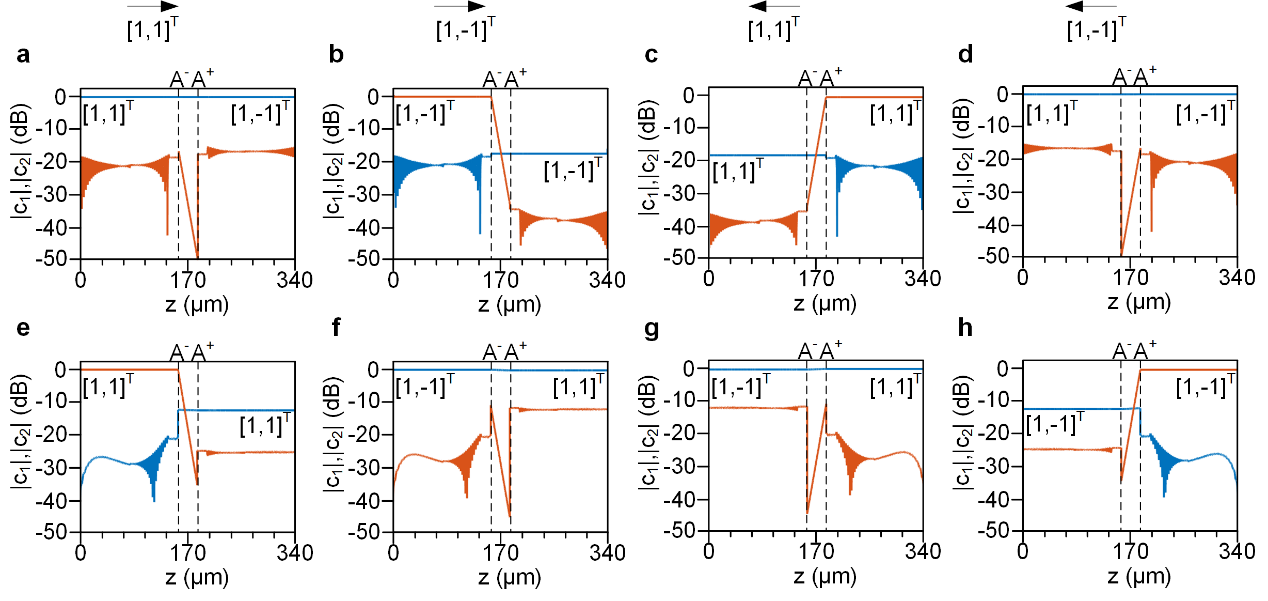


**Fig. S6 | Dynamics of the evolution trajectories for TE and TM polarizations after the length is doubled.** Coefficients |c_1_| and |c_2_| versus the propagation distance, z, for (**a**-**d**) TE and (**e**-**h**) TM polarizations.

Figure S6 shows the dynamics of the evolution trajectories of TE and TM polarizations when the device length is doubled. The results indicate that the mode crosstalk decreases as the device length is prolonged to ensure the modes evolve in a more adiabatic manner. However, from the perspective of device performance, while a longer device helps reduce crosstalk, it comes at the cost of increased fabrication complexity and larger device size, which can hinder high-density photonic integration.

**Supplementary Note 4: Fabrication details and measurement scheme**


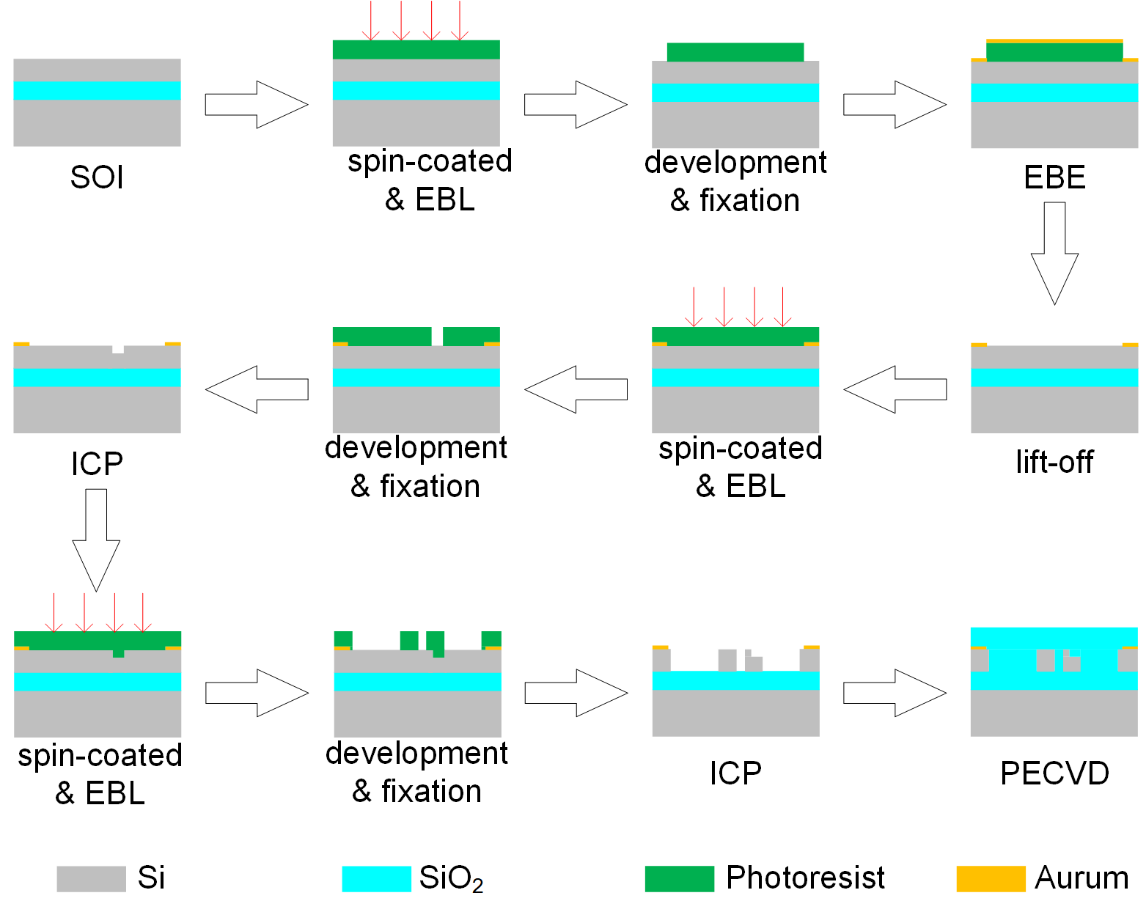


**Fig. S7 | Fabrication process of the samples.**

Figure S7 shows the fabrication process of our device samples with a combination of three-step electron-beam lithography (EBL), inductively coupled plasma (ICP) etching, electron-beam evaporation (EBE), and plasma-enhanced chemical vapor deposition (PECVD).

Firstly, an SOI wafer was successively cleaned in ultrasound bath in acetone, isopropyl alcohol and deionized water, and then was dried under nitrogen flow. A 20-nm-thick Aurum layer with a 5-nm-thick Chromium adhere layer, was fabricated as the alignment marks by a first-step EBL, EBE and lift-off process. Photoresist was spin-coated onto the wafer surface and was patterned by EBL, which was followed by development and fixation. The Aurum marks were successively deposited by EBE, and the final alignment marks were formed by lift-off process. Secondly, the partially-etched layer of the L-shaped silicon waveguides was fabricated by using a second-step EBL and ICP etching. The photoresist was patterned by use of EBL, followed by ICP etching in which the etching time is precisely controlled to define the etching depth of 120 nm for the partially-etched layer. Thirdly, the fully-etched layer of the L-shaped silicon waveguides was fabricated by using a third-step EBL and ICP etching. The ICP etching time was controlled to define the 340-nm-height fully-etched layer after the photoresist was patterned by EBL. Finally, a 1-μm-thick SiO_2_ layer is deposited by PECVD, to cover the entire sample for the optical field symmetry and structural protection.


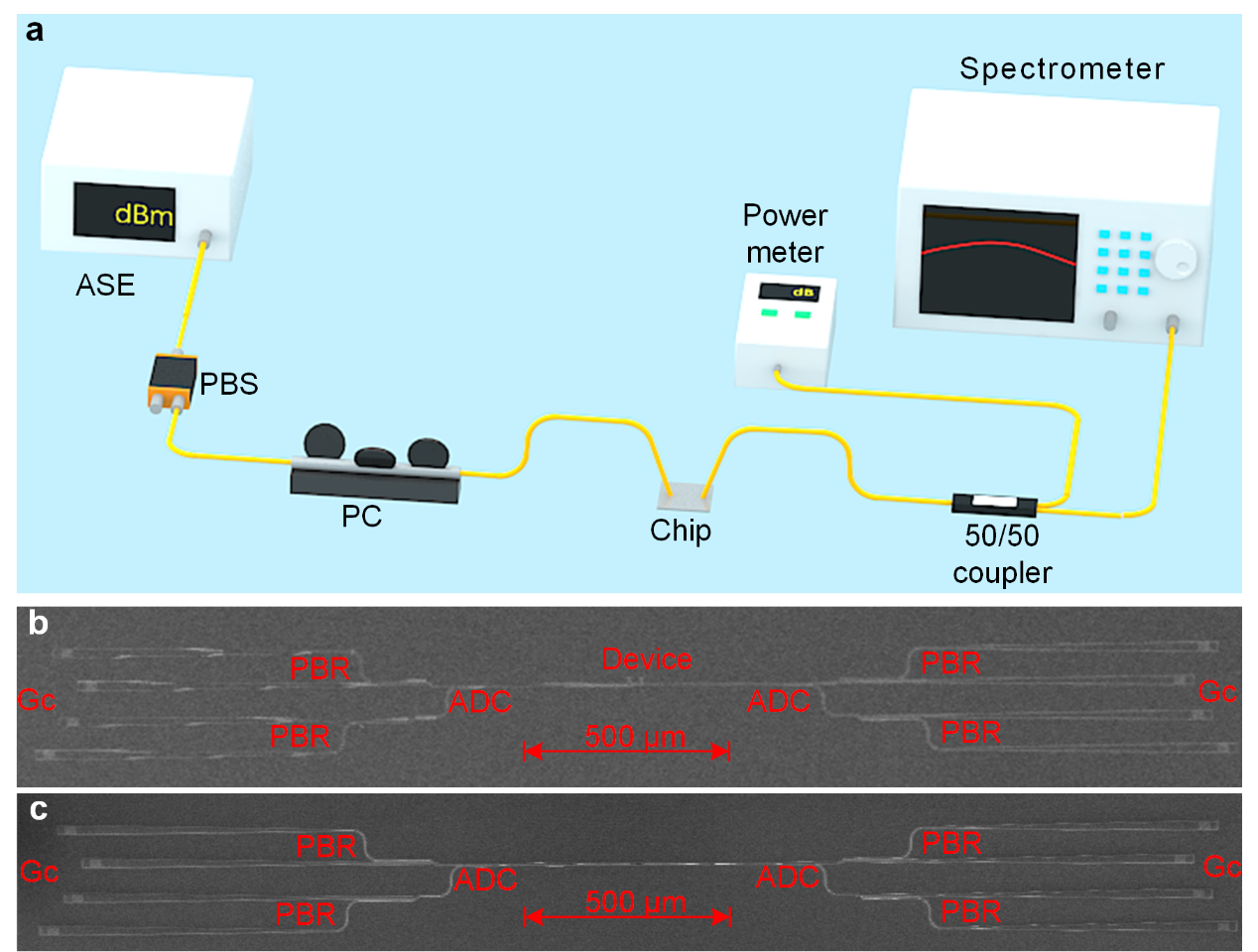


**Fig. S8 | Experimental demonstration.** **a** The experimental configuration. **b** The SEM image of the fabricated sample consisting of the double-coupled silicon waveguides, GCs, ADCs and PBRs. **c** The SEM image of the control device without the double-coupled silicon waveguides.

Figure S8 presents the experimental setup for measuring the transmittance of the fabricated device. The near infrared light is provided by an amplified spontaneous emission (ASE) broadband light source (spectral range 1525 to 1600 nm). The polarization of the incident light is adjusted by polarization beam splitter (PBS) and polarization controller (PC) before light is coupled into the grating coupler (GC) through the fiber. The emergent light from the SOI chip is measured by the optical power meter and spectrometer. The optical power meter is used to adjust the angle between the fiber and the GC so as to maximize the coupling efficiency between them.

TE_1_ (TM_1_) mode separates from the straight bus waveguide and is converted into TE_0_ (TM_0_) mode by asymmetrical directional coupler (ADC). The polarization beam splitter and rotator (PBR) is used to generate or split TE_0_ and TM_0_ modes at the input and output ports. Figure S9 presents the simulated results of the PBR. Figure S9a shows the intensity distributions of the electric field when TE_0_ mode inputs from port 1. The electric field intensity is close to 0 at port 3, indicating that only TE_0_ mode outputs at port 2. Figure S9b shows the intensity distributions of the electric field when TM_0_ mode inputs from port 1. The electric field intensity is close to 0 at port 2, and TM_0_ mode is converted into TE_0_ mode that outputs at port 3. Subsequently, the TE_0_ modes from ports 2 and 3 are output to the detectors through the GCs. On the contrary, when light is coupled into the port 2 (port 3) with TE_0_ mode through the GCs, it transforms into the TE_0_ (TM_0_) mode that outputs from port 1. Figures S9c, d show the transmittance of the TE_0_ mode at port 2 and port 3, when the TE_0_ and TM_0_ modes input from port 1, respectively. The extinction ratio is defined as the ratio of the energy at the desired port to the energy at the undesired port. The estimated extinction ratios are larger than 35 dB over 1500−1600 nm, no matter when the TE_0_ and TM_0_ mode is injected, indicating that the PBRs hardly affect the measured transmittance of the double-coupled silicon waveguides.

The control device without the double-coupled silicon waveguides is used to evaluate the loss arising from ADCs, GCs and PBRs. The loss differences in Figs. S8b, c can be used to extract the loss for different TE (TM) modes in the double-coupled silicon waveguides.


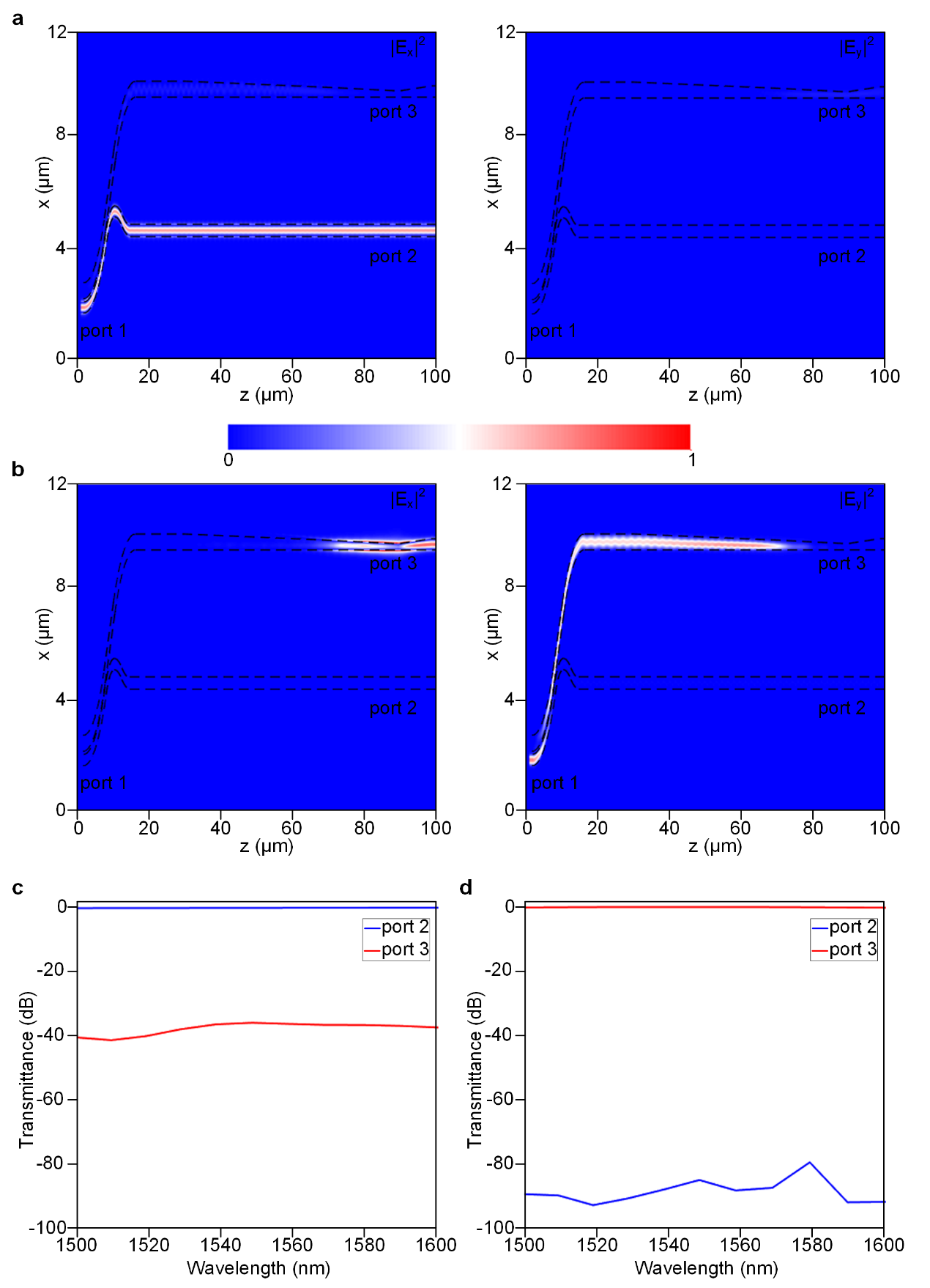


**Fig. S9 | Simulated results.** **a**, **b** Field intensity distributions of $\left|E_{x}\right|^{2}$ and $\left|E_{y}\right|^{2}$ along the propagation direction, when (**a**) TE_0_ and (**b**) TM_0_ modes input from port 1. **c**, **d** Transmittance of the TE_0_ mode at port 2 and port 3, when (**c**) TE_0_ and (**d**) TM_0_ modes input from port 1.


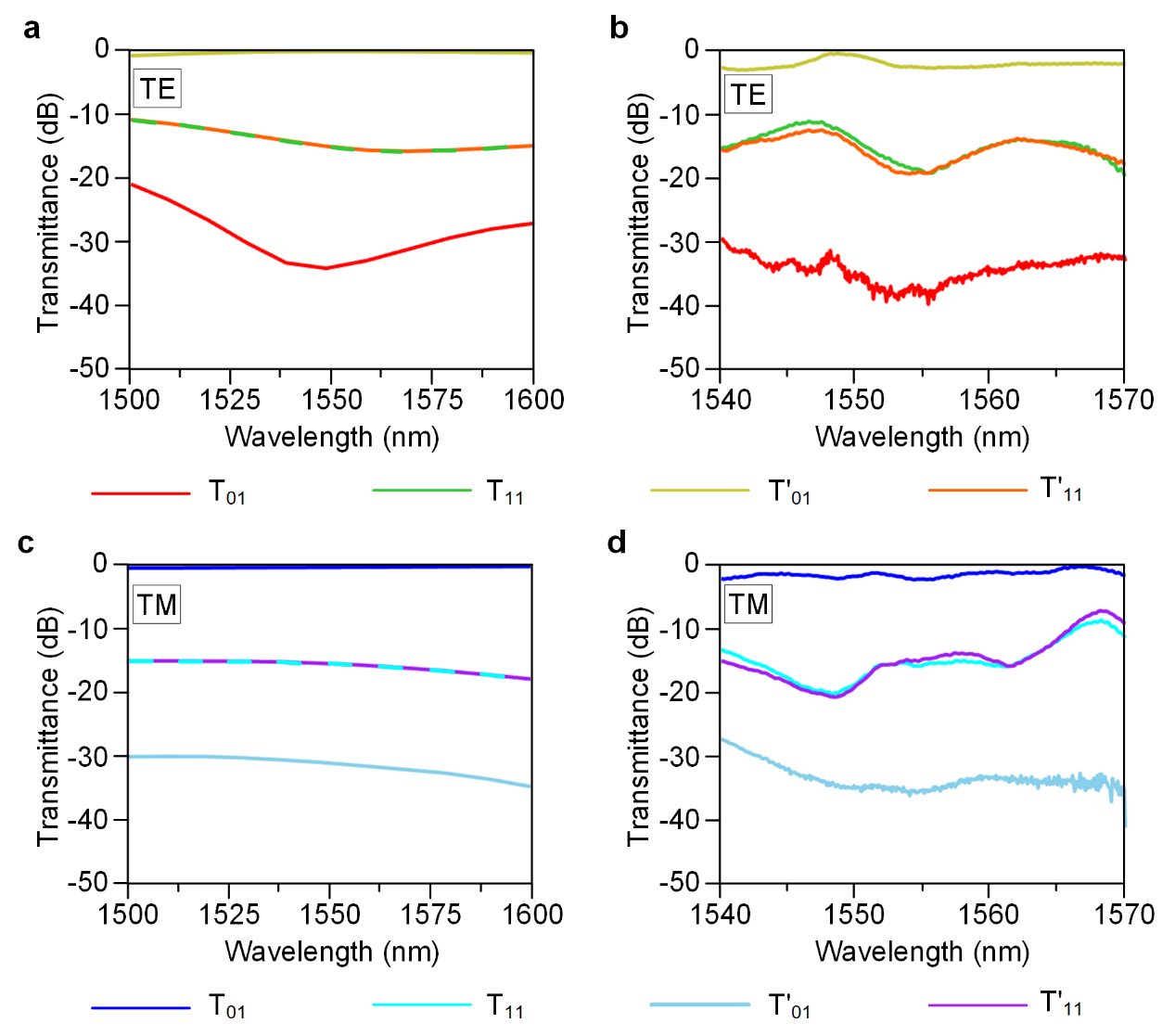


**Fig. S10 | Transmittance spectra.** **a**, **c** Simulated transmittance spectra at the output port over the wavelength range of 1500–1600 nm. For TE_0_ and TE_1_ mode (**a**), TM_0_ and TM_1_ mode (**c**). **b**, **d** Experimental transmittance spectra at the output port over the wavelength range of 1540–1570 nm. For TE_0_ and TE_1_ mode (**b**), TM_0_ and TM_1_ mode (**d**).

**Supplementary Note 5: Alignment error**


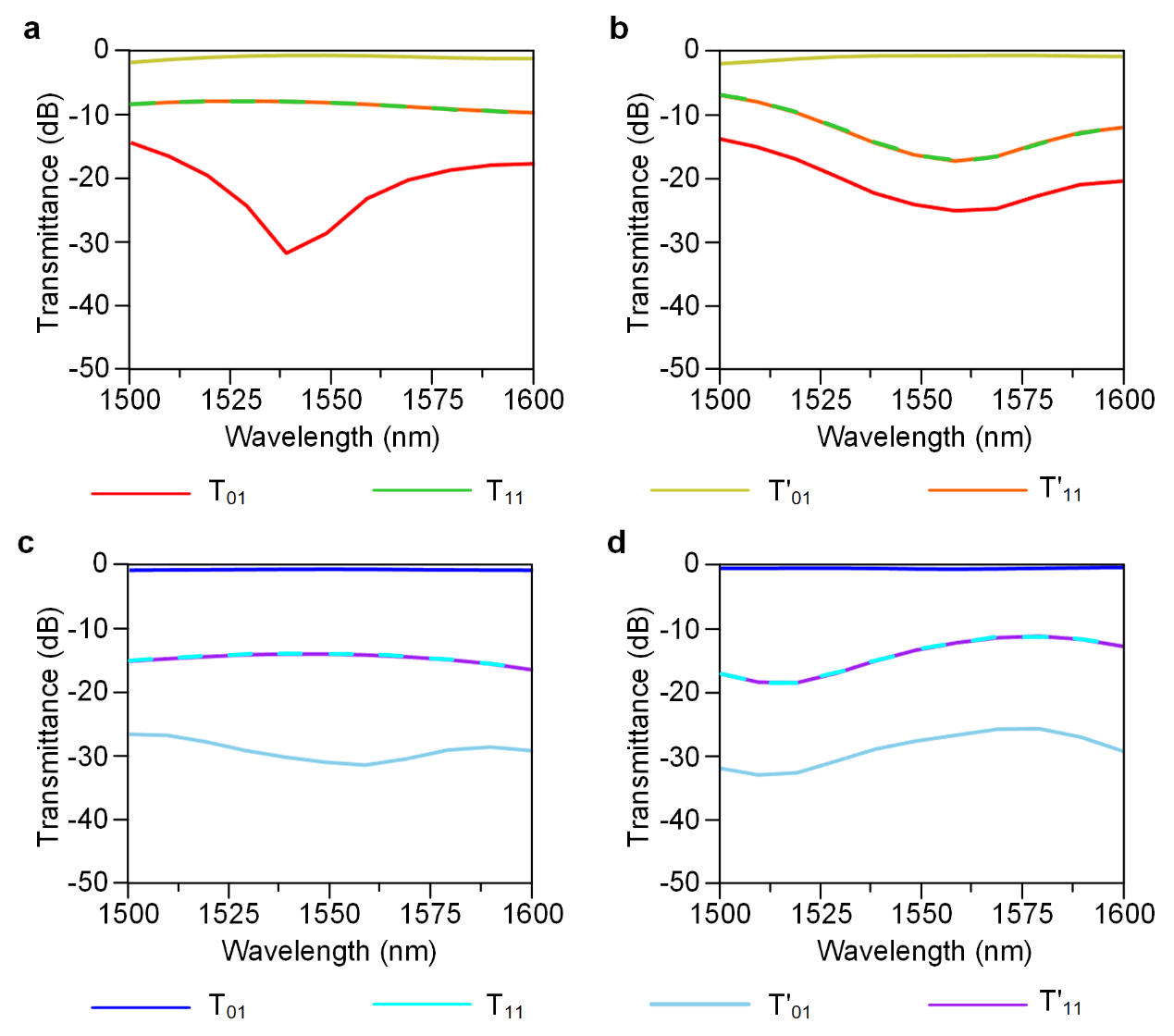


**Fig. S11 | Transmittance spectra.** Simulated transmission spectra of the double-coupled silicon waveguides, when TE_1_ (**a**, **b**) and TM_1_ modes (**c**, **d**) are injected. **a**, **c** indicates a positive alignment error, i.e., W_Ra_ is enhanced by 50 nm, and W_La_ is decreased by 50 nm, with respect to the baseline geometrical parameters. **b**, **d** indicates a negative alignment error, i.e., W_Ra_ is decreased by 50 nm, and W_La_ is enhanced by 50 nm, with respect to the baseline geometrical parameters.

If the alignment position of the second-step EBL and the third-step EBL are not completely consistent, W_Ra_ and W_La_ may deviate from the predesigned values. Figure S11 demonstrates that the double-coupled silicon waveguides can maintain well transmission spectra in the wavelength range of interest, even if the alignment errors are within the range of ±50 nm.

**Supplementary Note 6: Chiral transport for polarization diversity in three-coupled L-shaped waveguides**

**
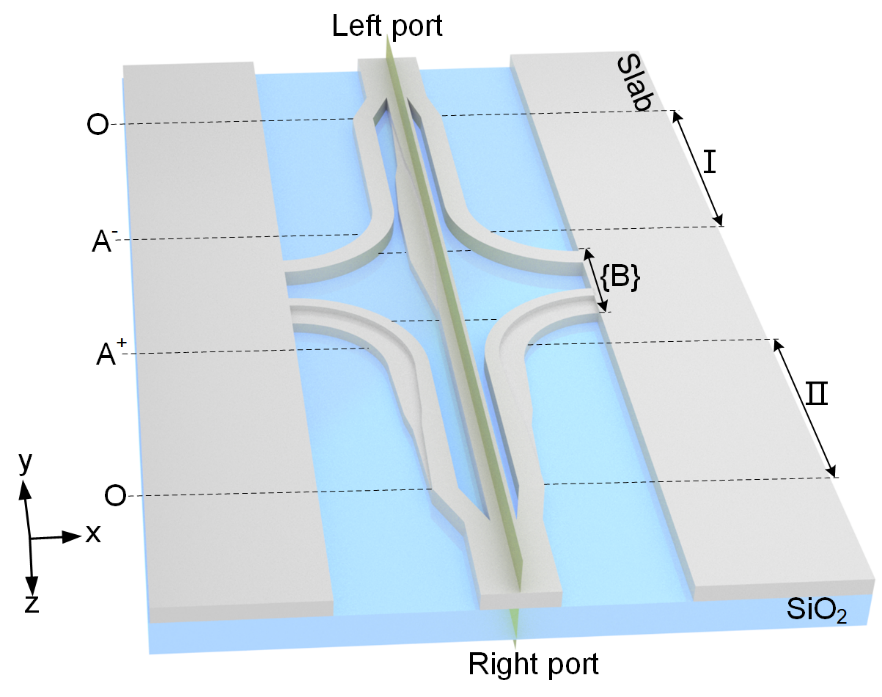
**

**Fig. S12 | Schematic diagram of three-coupled L-shaped waveguides.**

This structure differs from the double-coupled waveguides shown in Fig. 1c of the main text by the addition of an additional right waveguide and a slab waveguide on the left side. As a result, the new structure is symmetric with respect to the green plane shown in Fig. S12. Additionally, the widths of bus waveguides at both ends are increased from 900 nm to 1350 nm to support TE_2_ and TM_2_ modes. This modification enables polarization-controlled chiral transmission between TE_0_ (TM_0_) and TE_2_ (TM_0_). Figures S13 and S14 show the simulated field distributions and transmittance spectra. The simulation results indicate that the output mode is TE_2_ (TE_0_) and TM_0_ (TM_2_) modes for the left (right) port input with TE_0_ and TM_0_ modes, respectively. It should be noted that polarization diversity in even higher-order systems could be achieved by using more L-shaped coupled waveguides, in principle.

**
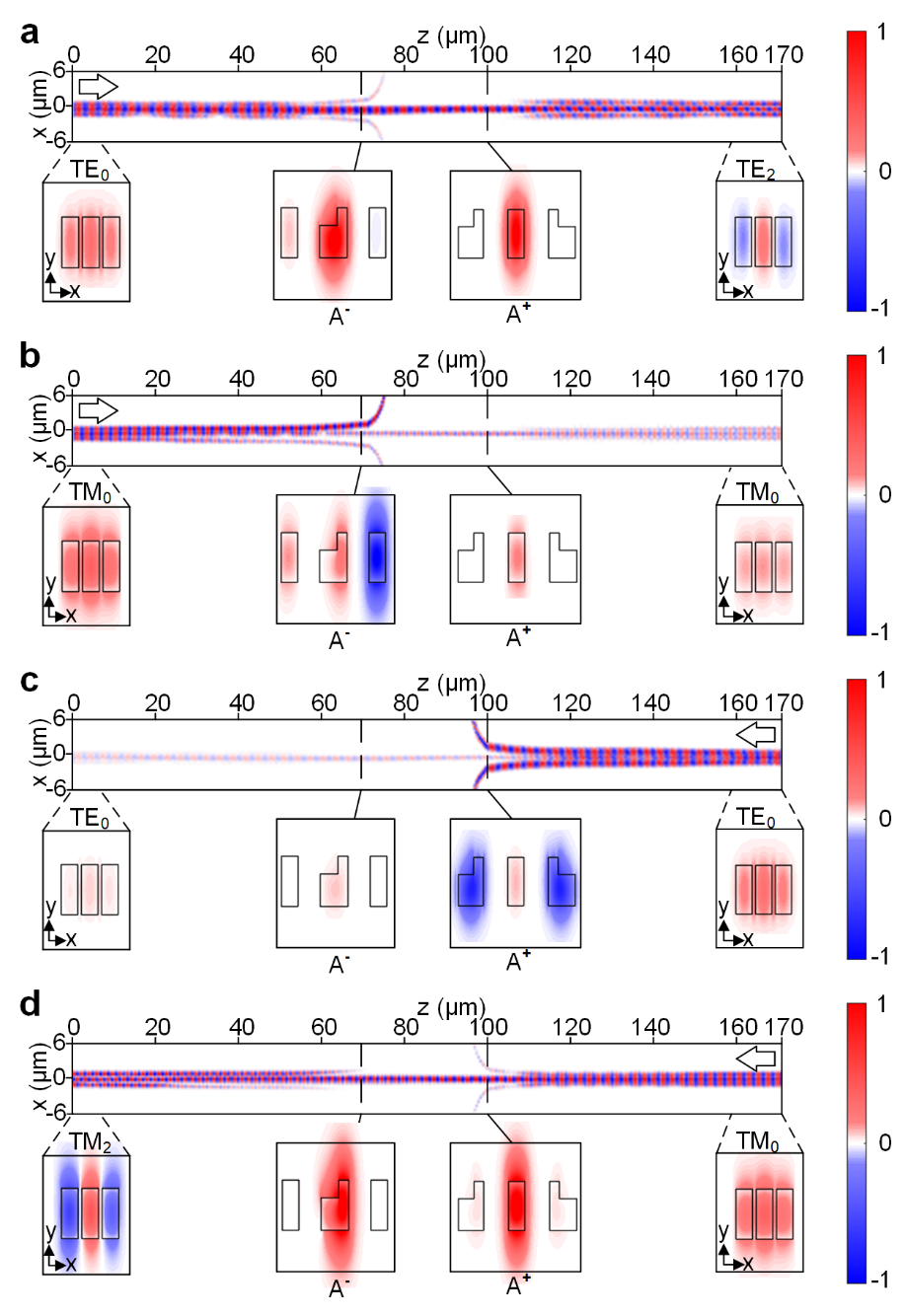
**

**Fig. S13 | Simulated field distributions.** **a**, **c** Field distributions of E_x_, when TE_0_ mode inputs from the left (**a**) and right (**c**) ports, respectively. **b**, **d** Field distributions of E_y_, when TM_0_ mode inputs from the left (**b**) and right (**d**) ports, respectively.


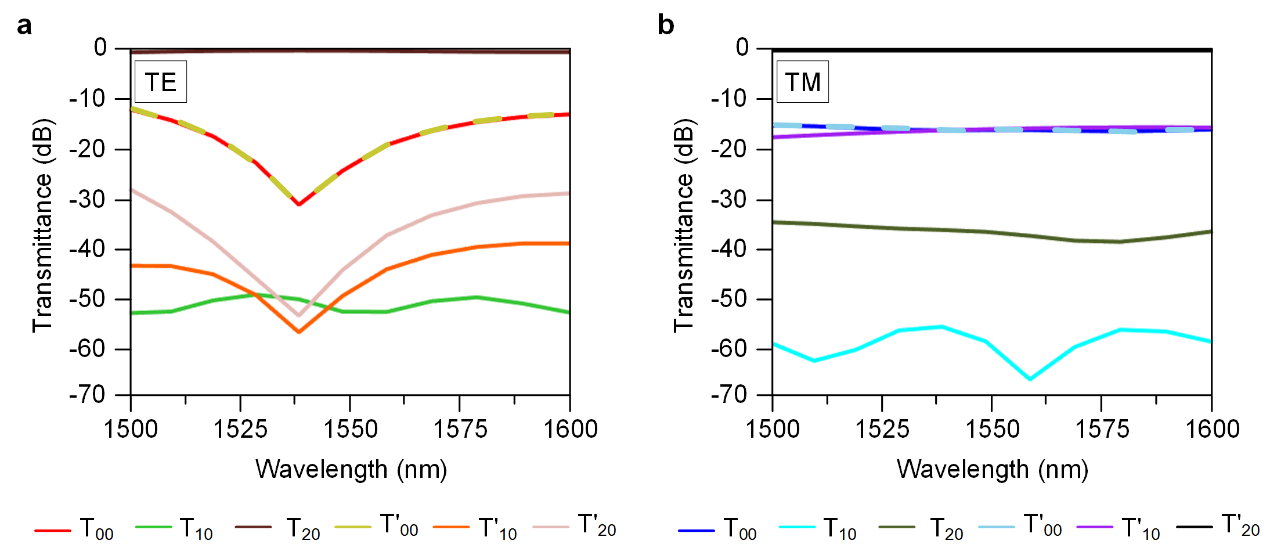


**Fig. S14 | Transmittance spectra.** **a, b** Simulated transmittance spectra at the output port over the wavelength range of 1500–1600 nm with TE_0_ (**a**) and TM_0_ (**b**) injection.

**Supplementary Note 7: Shortened device**


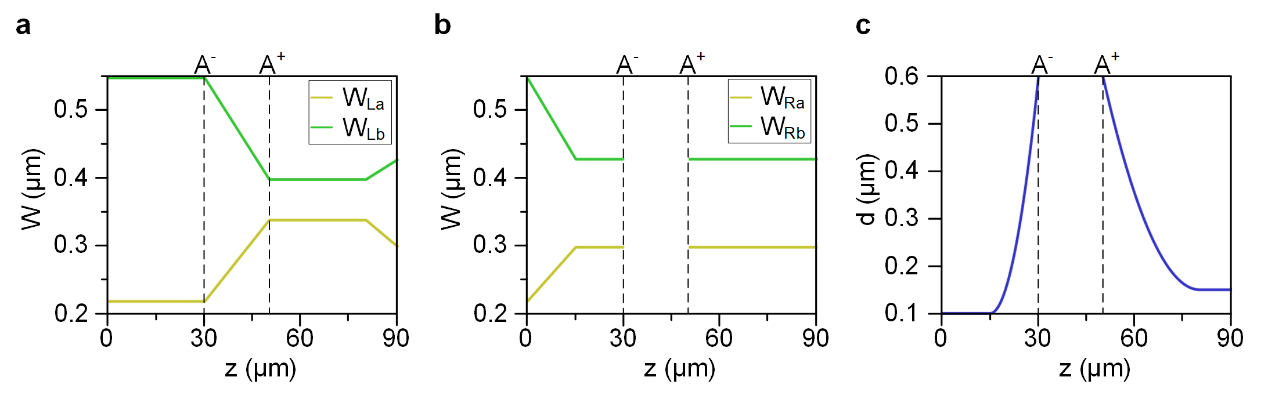


**Fig. S15 | Structural parameters of the shortened device.** **a** Width of the left waveguide; **b** Width of the right waveguide; **c** Gap distance, versus z.

When alignment errors are not considered, simulations using the structural parameters shown in Fig. S15 yield the transmission spectra presented in Fig. S16. It can be observed that, after altering the structural parameters, the device length reduces from the 170 nm length in the main text to 90 nm, while still achieving the expected results.


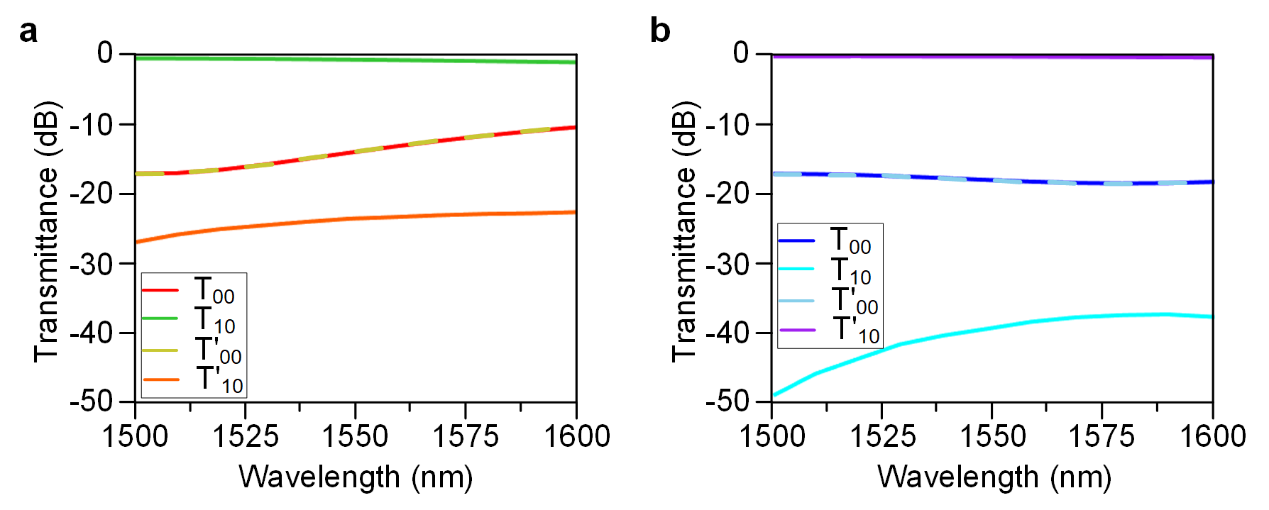


**Fig. S16 | Transmittance spectra.** Simulated transmittance spectra for TE_0_ (**a**) and TM_0_ (**b**) mode is injected.
